# A newly high alkaline lipase: an ideal choice for application in detergent formulations

**DOI:** 10.1186/1476-511X-10-221

**Published:** 2011-11-28

**Authors:** Slim Cherif, Sami Mnif, Fatma Hadrich, Slim Abdelkafi, Sami Sayadi

**Affiliations:** 1 Laboratoire des Bioprocédés Environnementaux, Pôle d'Excellence Régional (PER, AUF), Centre de Biotechnologie de Sfax, Université de Sfax, B.P. "1177", Sfax 3018, Tunisia

## Abstract

**Background:**

Bacterial lipases received much attention for their substrate specificity and their ability to function in extreme environments (pH, temperature...). Many staphylococci produced lipases which were released into the culture medium. Reports of thermostable lipases from *Staphylococcus *sp. and active in alkaline conditions are not previously described.

**Results:**

A newly soil-isolated *Staphylococcus *sp. strain ESW secretes an induced lipase in the culture medium. The effects of temperature, pH and various components in a detergent on the activity and stability of *Staphylococcus *sp. lipase (SL1) were studied in a preliminary evaluation for use in detergent formulation solutions. The enzyme was highly active over a wide range of pH from 9.0 to 13.0, with an optimum at pH 12.0. The relative activity at pH 13.0 was about 60% of that obtained at pH 12.0. It exhibited maximal activity at 60°C. This novel lipase, showed extreme stability towards non-ionic and anionic surfactants after pre-incubation for 1 h at 40°C, and relative stability towards oxidizing agents. Additionally, the crude enzyme showed excellent stability and compatibility with various commercial solid and liquid detergents.

**Conclusions:**

These properties added to the high activity in high alkaline pH make this novel lipase an ideal choice for application in detergent formulations.

## Background

Lipases (EC 3.1.1.3) represent an important group of biotechnologically valuable enzymes [1-3]. They are widely distributed in nature. Although lipases have been found in many species of animals, plants, bacteria, yeast, and fungi, the enzymes from microorganisms are the most interesting because of their potential applications in various industries such as food, dairy, pharmaceutical, detergents, textile, biodiesel, and cosmetic industries and in synthesis of fine chemicals, agrochemicals, and new polymeric materials [4-6]. Detergent industries are the primary consumers of enzymes, in terms of both volume and value [7]. The use of enzymes in detergents formulations enhances the detergents ability to remove tough stains and making the detergent environmentally safe. Nowadays, many laundry-detergent products contain cocktails of enzymes including proteases, amylases, cellulases, and lipases [8]. As a detergent additive, the increasing usage of alkaline lipase is mainly due to its affiliation with the nonphosphate detergents. Ideally, alkaline lipases in a detergent should have high activity and stability over a broad range of temperature and pH, and should also be compatible with different components in a detergent including metal ions, surfactants and oxidants [9]. Bacterial lipases received much attention for their substrate specificity and their ability to function in extreme environments. Many staphylococci produce lipases which are released into the culture medium. Reports of thermostable lipases from *Staphylococcus *sp. and active in alkaline conditions are not previously described. Also, practical applications of staphylococcal enzymes may be limited due to relatively lower stabilities and catalytic activities under conditions that characterise industrial processes: high temperatures, extremes of pH values or non-aqueous solvents. In the past years, intense efforts have been focused on the engineering of enzymes with altered properties or better performance for practical applications. Therefore, screening of new microorganisms with lipolytic activities could facilate the discovery of novel lipases. Recently we isolated and optimized the production of lipase from a newly *staphylococcus *sp strain ESW (unpublished data). After optimization of culture conditions and medium composition, biochemical properties of crude lipase were investigated. Within this context, we report the characterisation of a thermoactive, alkaline and detergent-stable lipase (SL1) from a newly isolated *staphylococcus *sp strain ESW, and investigate its compatibility with various surfactants, oxidizing agents, commercial liquid and solid detergents to evaluate its potential for detergent formulation.

## Methods

### Chemicals

Tributyrin (99%, puriss) and benzamidine were from Fluk (Buchs, Switzerland); tripropionin (99%, GC) was from Jansen (Pantin, France); phosphatidylcholine, sodium deoxycholic acid (NaDC), sodium taurodeoxycholic acid (NaTDC), Tween 80, yeast extract and ethylene diamine tetraacetic acid (EDTA) were from Sigma Chemicals (St. Louis, USA); β-mercaptoethanol was from Merck (Darmshtadt, germany); all other detergents used (Ariel, Axion and Omino Bianco) were purchased locally; gum Arabic was from Mayaud Baker LTD (Dagenham, United Kingdom); pH-stat was from Metrohm (Zofingen, Switzerland).

### Screening of lipolytic microorganisms

Initial screening of lipolytic microorganisms from various biotopes was carried out using a plate assay in a medium containing triacylglycerol and the fluorescent dye Rhodamine B [10,11]. The solid medium contains 1‰ olive oil, 1% nutrient broth, 1% NaCl, 1.5 g agar and 1‰ Rhodamine B. The culture plates were incubated at 37°C, and colonies giving orange fluorescence halos around them, upon UV irradiation, were regarded as putative lipase producers [12]. After extensive screening of lipase producers, only one bacterial colony, isolated from an hydrocarbure contaminated soil continued to give a positive signal when commercial detergent (1%) was added to the solid medium described above. The identification of this strain has been kindly determined by Dr. Abdelhafedh Dhouib (Centre de biotechnologie de Sfax, Tunisia). The biochemical properties and the morphological aspect of this microorganism showed 100% identity to *Staphylococcus *strain.

### Media and culture conditions

*Staphylococcus *sp. was incubated overnight at 37°C and 200 rpm in 1-liter-shaking flasks with 100 mL of Luria-Bertani broth medium composed of (g/L): peptone, 10.0; yeast extract, 5.0; NaCl, 5.0; 1% olive oil; pH 7.0. (medium A). Overnight *Staphylococcus *sp. cultures used as inocula were cultivated in 1-liter shaking flasks with 100 ml of the medium A supplemented with 1% olive oil (medium B). The culture was incubated aerobically during 36 h on a rotary shaker set at 160 rpm and at a temperature of 37°C. The cultures were centrifuged at 12 000 rpm for 15 min at 4°C, and the cell-free supernatants were used for estimation of lipase activity. Growth was followed by measuring the cultures optical density (OD) at 600 nm.

### Lipase activity determination

The lipase activity was measured titrimetrically at pH 12 and 60°C with a pH-stat under standard conditions using tributyrin (0.25 mL) in 30 ml of 2.5 mM Tris-HCl pH 12, 2 mM CaCl_2_, 1 mM NaDC or olive oil emulsion (10 mL in 20 mL of 9‰ NaCl pH 12, 2 mM CaCl_2_, 2 mM NaDC) as substrate. Lipase activity was also measured at pH 7 and 37°C using TC_3 _as substrate (0.25 mL TC_3_) in 30 mL of 2.5 mM phosphate buffer pH 7, 2 mM CaCl_2_. The olive oil emulsion was obtained by mixing (3 × 30 s in a Waring blender) 10 mL of olive oil in 90 ml of 10% GA. When measuring SL1 lipase activity in the absence of CaCl_2_, EDTA or EGTA was added to the lipolytic system. Lipolytic activity was expressed as units. One unit corresponds to 1 μmol of fatty acid released per minute.

### Determination of substrate specificities

Activity of the crude lipase towards different triacylglycerols was determined by pH-stat assay under optimal conditions (pH 12.0 and 60°C). The triacylglycerols triacetin (TC_2_), tripiopionin (TC_3_), tributyrin (TC_4_), trioctanoin (TC_8_), and triolein (C_18_) at a final concentration of 10 mM. The triolein was emulsified immediately before use in 10% gum Arabic solution as described previously [13].

### Effect of pH and temperature on SL1 activity and stability

SL1 activity was tested in various buffers at different pH (5-13) at 60°C. The pH stability of the lipase was determined by incubating the enzyme at different pH (3-12) for 24 h at room temperature. The residual activity was determined, after centrifugation, under standard assay method [14].

The optimum temperature for the SL1 activity was determined by carrying out the enzyme assay at different temperatures (25-65°C) at pH 12. The effect of temperature on lipase stability was determined by incubating the enzyme solution at different temperatures (30-60°C) for 60 min. The residual activity was determined, after centrifugation, under standard assay method.

### Effects of metal ions on enzyme activity

The effect of various metal ions on lipase activity was investigated by adding divalent metal ions (Ca^2+^, Mn^2+^, Zn^2+^, Cu^2+^, Ba^2+^, Mg^2+^) to the reaction mixture. The activity of the crude enzyme without metallic ions was considered as 100%.

### Effect of surfactants and detergents on enzyme stability

The suitability of *Staphylococcus *sp crude enzyme as a detergent additive was determined by testing its stability in the presence of some surfactants such as SDS (sodium dodecyl sulphate), Triton X-100, Tween 20, and oxidizing agents such as hydrogen peroxide (H_2_O_2_) and sodium perborate (NaBO_3_). Crude enzyme containing alkaline lipase, at 15 U/mL was incubated with different additives for 1 h at 40°C and then the residual enzyme activities were determined under standard assay conditions. The activity of the crude enzyme, incubated under similar conditions without any additive was taken as 100%. The compatibility of the ESW enzymatic preparation with commercial solid and liquid laundry detergents was also studied. The solid detergents tested were Dixan (Henkel, Spain), Nadhif (Henkel-Alki, Tunisia), Ariel (Procter and Gamble, Switzerland) and Axion (Colgate-Palmolive, France). The liquid detergents tested were Dixan (Henkel, Spain), Nadhif (Henkel-Alki, Tunisia) and Lav+ (Best LAV, Tunisia). Solid detergents were diluted in tap water to give a final concentration of 5 mg/l and liquid detergents were diluted 100-fold to simulate washing conditions. The endogenous enzymes contained in these detergents were inactivated by heating the diluted detergents for 30 min at 80°C prior to the addition of the ESW crude enzyme. Crude enzyme containing alkaline lipase, at 15 U/mL, was added to solid detergents diluted in tap water and incubated in various detergent solutions for 1 h at different temperatures, and then the residual enzyme activity was determined under standard assay conditions. To allow further comparison, the effect of surfactants, commercial detergents and oxidizing agents on a commercial lipase stability (Lipolase^®^, marketed by Novo Nordisk, Denmark), was also studied under the same experimental conditions. The enzyme activity of the control sample (without any detergent), incubated under the same conditions, was taken as 100%.

### Determination of protein concentration

Protein concentration was determined as described previously by Bradford [15] using bovine serum albumin (BSA) as the standard.

### Statistical analyses

All results are expressed as the mean ± standard deviation (± SD). The experiment was conducted at least 3 times, and each treatment had 3 replicates. Thus, for most data points, the *n *= 3. The SAS System for Windows, V8 (SAS Institute, Gary, NC) was used for statistical evaluations. Means ± S.D. were calculated for normalizing the control as 100%. Differences among treatment and control groups were tested by one-way analysis of variance (ANOVA), followed by pair-wise comparisons between groups using Tukey's test. Differences at *p *< 0.05 were considered significant.

## Results and Discussion

### Production of lipase

The medium B (100 mL) was incubated with different amounts of inoculum from the overnight *Staphylococcus *sp. culture. The maximum lipase production (15 U/mL of culture medium) was obtained after 30-h incubation, with an initial absorbance (OD) measured at 600 nm of 0.2 and an inoculum size of 3 × 10^8 ^cells/L. Our results show that the time course of lipase production followed at 37°C with cell growth. The lipase activity was observed to start soon after incubation and reached the maximum (30 U/mL) at the end of the exponential phase corresponding to 30 h of cultivation (data not shown). Lipases are generally produced using carbon source such as oils, fatty acids, glycerol or tweens in the presence of an organic nitrogen source [16]. In fact, the production of SL1 is induced by the presence of long chain triacylglycerols (like olive oil).

### Interfacial activation of SL1

**Figure 1:**
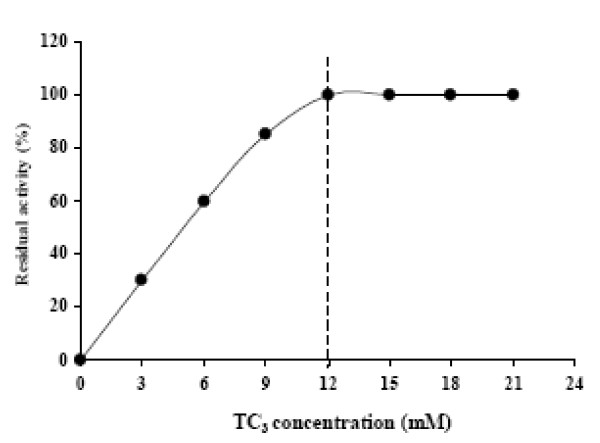
**Hydrolysis rate of TC_3 _by SL1 as function of substrate concentration**. The TC_3 _solutions were systematically prepared by mixing (3 × 30 s in warring blender) a given amount of TC_3 _in 30 mL of 0.3% GA and 0.15 M NaCl. The release of propionic acid was recorded continuously at pH 7 and 37°C using a pH-stat. The CMC of TC_3 _(12 mM) is indicated by vertical dotted lines.

The hydrolysis rate of TC_3 _emulsified in 0.33% GA and 0.15 M NaCl by SL1 as a function of substrate concentration shows a normal Michaelis-Menten dependence of the activity on the substrate concentration (Figure 1). The interfacial activation cannot be taken as the unique criterion required distinguishing lipases from esterases [17]. Lipases are defined as a family of enzymes able to hydrolyse long-chain triacylglycerols independently of the presence, or the absence, of the interfacial activation phenomenon. Here, we can say that SL1, which hydrolyses efficiently olive oil, is a true lipase.

### Substrate specificity

**Figure 2:**
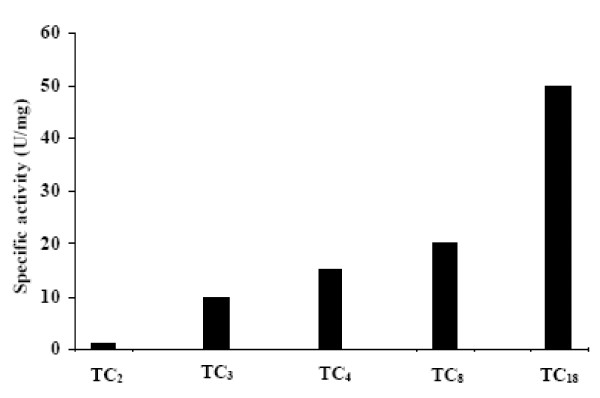
**Chain length selectivity of SL1 on various triacylglycerols**. Lipase activities are expressed as the percentage of that of TC_18_, which was taken as 100%.

The enzymatic activity of lipases is very sensitive to the physical state of the substrate. In fact, the chain length selectivity constitutes an important difference between staphylococcal lipases. Both *Staphylococcus aureus *lipase and *Staphylococcus hyicus *lipase have a strong preference for short-chain substrates [18], whereas *Staphylococcus similans *lipase [19] and native *Staphylococcus xylosus *lipase [20] hydrolyse triacyglycerols irrespective of their chain length. Lipolytic activities of SL1 were checked towards several triacylglycerols substrates (Figure 2). The highest activity of SL1 was recorded on TC_18 _(specific activity of 50 U/mg). A specific activity of 20, 15 and 10 U/mg was measured on TC_8_, TC_4 _and TC_3 _respectively, whereas no activity was detected on TC_2_. These results strongly show that this enzyme acts as a true lipase.

### Effects of pH and temperature on SL1 activity and stability

**Figure 3:**
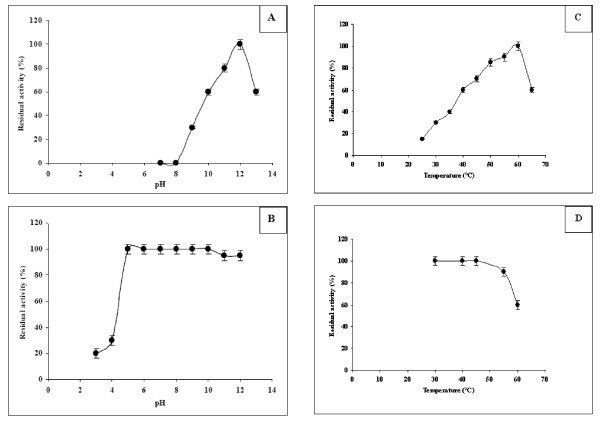
**pH effect on enzyme activity (A) and stability (B) of SL1**. Optimal pH was determined with olive oil emulsion at 60°C under the standard conditions. Stability was analysed after preincubating the crude enzyme for 24 h in different buffer solutions at various pH ranging from 3 to 12. Temperature effect of on SL1 activity (C) and stability (D). For temperature stability the crude enzyme was preincubated at different temperatures for 1 h and the remaining activity was measured under the standard conditions. The activity of the enzyme before incubation was taken as 100%. Values presented are the mean of triplicate analyses.

The importance of alkaline and thermostable lipases for different applications has been growing rapidly. A great deal of research is currently going into developing lipases, which will work under alkaline conditions as fat stain removers. The activity of SL1 was investigated at different pH using olive oil emulsion as substrate. Unlike all previously described staphylococcal lipases [21], our results show that SL1 remains active at a pH range of 9.0-13.0, with an optimum at pH 12.0 (Figure 3A). This result recalls that observed in the case of *staphylococcus aureus *lipase. In fact, the optimum pH of lipase activity was found to be ranged between pH 8.0 and 10.0 [22]. In the pH stability study, the lipase is stable at abroad range of pH values between pH 5.0-12.0 after 24 h incubating (Figure 3B). Moreover, this behaviour is in accordance with other reports in the literature, which lipase was found to be stable between pH 5.0 and 12.0. [22]. Lipases active and stable in alkaline media are very attracting, for example, lipase produced by *Acinetobacter radioresistens *has an optimum pH of 10.0 and it was stable over a pH range of 6.0-10.0; this enzyme has a great potential for application in the detergent industry [23,24]. The lipase activity was also determined at different temperatures under optimal conditions (Figure 3C). In contrast to the majority of staphylococcal lipases described so for [3,19,25], the SL1 activity increased significantly with increasing the temperature to reach its maximum value at 60°C. The thermostability of SL1 was also determined by measuring the residual activity after incubation of the pure enzyme at various temperatures (Figure 3D). In contrast to *Staphylococcus similans *lipase which is inactivated after a few minutes when incubated at 60°C [19,26], SL1 retained 90% or 60% of its activity after a 60 min incubation at 55 or 60°C, respectively. Thermostable and alkaline lipases are therefore highly attractive [24] to the synthesis of biopolymers and biodiesel and used for the production of pharmaceuticals, agrochemicals, cosmetics, and flavour [13].

### Effects of calcium and other metal ions on SL1 activity

Metal cations, particularly Ca^2+^, play important roles in influencing the structure and function of enzyme, and calcium-stimulated lipases have been reported [27]. Previously, it has been demonstrated that the activity of staphylococcal lipases may depend on the presence of Ca^2+ ^ions [19]. The effect of various Ca^2+ ^concentrations on the rate of hydrolysis of SL1 was studied. Our results showed that SL1 activity can be detected in the absence of Ca^2+^. A specific activity of 40 U/mg was measured in the presence of 10 mM of chelator such as EDTA or EGTA when using olive oil emulsion as substrate. In the absence of chelators, the specific activity of SL1 reached 50 U/mg at 2 mM CaCl_2 _(data not shown). The enzymatic activity of staphylococcal lipases is stimulated by Ca^2+^. It has been reported that the lipases from *P. glumae *[28] and *S. hyicus *[21,29], contain a Ca^2+^-binding site which is formed by two conserved aspartic acid residues near the active-site, and that binding of the Ca^2+ ^ion to this site dramatically enhanced the activities of these enzymes. The effects of various cations at a concentration of 2 mM on lipase activity were assessed (Table 1). Mg^2+ ^and Ba^2+ ^had little positive effect on lipase activity. Other metals such as Cu^2+^, Zn^2+ ^and Mn^2+ ^had no significant effect on enzyme activity in our study (Table 1).

**Table 1:** Effect of some metal ions (2 mM) on alkaline lipase activity of *Staphylococcus *sp ESW.

Metal ions	Relative lipase activity (%)
Ca^2+^	120 ± 1.5
Mg^2+^	108 ± 1.0
Zn^2+^	110 ± 1.7
Mn^2+^ Cu^2+^	100 ± 1.5 100 ± 1.0
Ba^2+^	100 ± 1.2
Control	100 ± 1.0

The activity was determined by incubating the crude enzyme in the presence of various metal ions (2 mM) under optimal conditions. The results are the mean ± S.D. of two independent experiments conducted in triplicate.

### Effect of surfactants and oxidizing agents on SL1 stability

In order to be effective during washing, a good detergent enzyme must be compatible and stable with all commonly used detergent compounds such as surfactants, oxidizing agents [30]. Lipase enzymatic preparation was pre-incubated for 1 h at 40°C in the presence of SDS, Tween 20 and Triton X-100 and the residual enzyme activities were assayed under standard assay conditions (Table 2). Lipase activity was highly stable in the presence of the non-ionic surfactants, retaining 100% of the original activity in the presence of 1% Triton X-100 and 1% Tween 20 after 1 h incubation at 40°C. Higher concentration of Triton X-100 (5%, v/v) caused a moderate inhibition, less than 10%. Furthermore, the crude lipase enzyme was highly stable in the presence of the strong anionic surfactant (SDS, 1%), retaining approximately 90% of the initial lipolytic activity after incubation for 1 h at 40°C. The stability towards SDS is important because SDS stable enzymes have been rarely reported. As shown in Table 2 the lipase was much more stable than the Lipolase^® ^in the presence of tween 20, triton X-100 and SDS. In addition, we investigated the effects of oxidizing agents (H_2_O_2 _and sodium perborate) on lipase stability pre-incubated for 1 h at 40°C. As shown in Table 2 lipolytic activity was little influenced by oxidizing agents. The crude lipase retained 80% of the initial activity in the presence of 0.5% (v/v) H_2_O_2 _and 0.2% (w/v) sodium perborate, respectively. Oxidizing reactions exerted by hydrogen peroxide and sodium hypochlorite are expected to occur in washing conditions. The stability of oxidations is an important characteristic required for an enzyme to be incorporated into a detergent. The oxidizing stability had been achieved by site-directed mutagenesis [31] and protein engineering [32] for many proteases [33] and the commercial detergent Lipolase^®^. As evidenced by the residual lipase activity (80% vs. 60%), our lipase showed higher resistance compared to Lipolase^®^, in the presence of strong oxidizing agent sodium hypochlorite (shown in Table 2). Thus, the high tolerance of oxidizing agents makes it an outstanding alkaline lipase of high commercial value.

**Table 2:** Stability of alkaline lipase of *Staphylococcus *sp ESW in the presence of various detergent components.

Detergent components	Concentration (%)	Residual activity (%)	
		SL1	**Lipolase**^**®**^
**None**		100 ± 1.0	100 ± 1.0

**Surfactants**			
Tween 20	1 (V/V)	100 ± 1.5	80 ± 1.0
Triton X-100	1 (V/V)	100 ± 1.7	60 ± 1.0
SDS	5 (V/V)	92 ± 1.2	40 ± 1.2
	0.1 (W/V)	90 ± 1.0	80 ± 1.0
	1 (W/V)	85 ± 1.5	70 ± 1.5
	5 (W/V)	80 ± 1.9	65 ± 1.0

**Oxidizing agents**			

H_2_O_2_	0.5 (V/V)	80 ± 1.1	60 ± 1.0
Sodium perborate	0.2 (V/V)	80 ± 1.5	60 ± 1.9

The crude enzyme preparation was incubated with different detergents components for 1 h at 40°C and the remaining activity was measured under standard assay conditions. The results are the mean ± S.D. of two independent experiments conducted in triplicate.

^a ^Initial activity = 15 U mL^-1 ^as 100% at 60°C and pH 12.0.

^b ^Initial activity = 100 U mL^-1 ^as 100% at 30°C and pH 9.0.

### Stability of SL1 with commercial solid and liquid detergents

**Figure 4:**
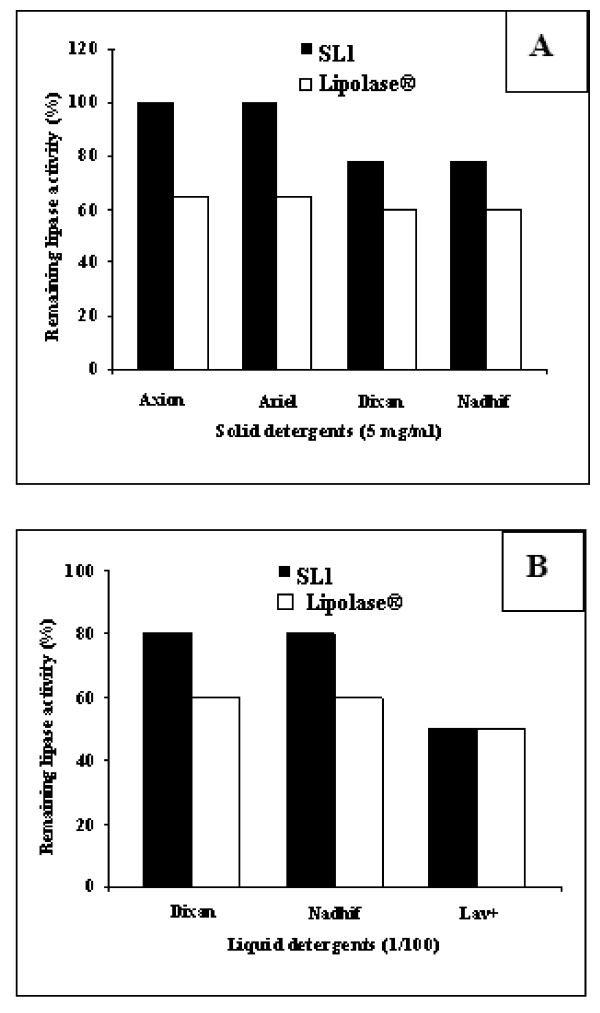
**Stability of the alkaline lipase from *Staphylococcus *sp ESW in the presence of various commercial solid (A) and liquid (B) detergents**. The alkaline lipase, at 15 U mL^-1^, was incubated with solid detergents at 5 mg mL^-1 ^in tap water (pH 10.0) and with liquid detergents diluted 100-fold in tap water (pH 9.0) for 1 h at 50°C. Commercial lipase (Lipolase^®^) was tested under the same conditions as ESW enzyme. The residual activity was measured under the standard enzyme assay condition. Enzyme activities of control samples without any detergent, incubated under the similar conditions, were taken as 100%. Values presented are the mean of triplicate analyses.

All the commercial detergents contain hydrolytic enzymes and these enzymes-based detergents known as "green chemicals" find a wide range of applications in laundry, dishwashing, textile and other related industries [34]. In order to check the compatibility with liquid and solid detergents, the crude enzyme was pre-incubated in the presence of various commercial laundry detergents for 1 h at 50°C. Commercial lipase (Lipolase^®^) was used under the same conditions as SL1. The solid detergents were diluted in tap water to a final concentration of 5 mg/mL and the liquid detergents were diluted 100-fold to simulate washing conditions. The data presented in Figure 4A show that the lipolytic preparation is extremely stable towards all solid detergents tested at 50°C, retaining 100% of its activity in the presence of Axion and Ariel and more than 80% with Dixan and Nadhif after 1 h incubation. Interestingly, SL1 was more stable than the commercially lipase used (Lipolase^®^), which retained only 65% and 60% of its activity after 1 h incubation at 50°C in the presence of Axion or Ariel and Dixan or Nadhif, respectively. On the other hand, the data presented in Figure 4B show that SL1 is extremely stable in the presence of all liquid detergents tested. The crude enzyme retained 80% of its activity in the presence of Dixan and Nadhif after 1 h incubation at 50°C. However, commercial lipase (Lipolase^®^) retained only 60% of its activity in the presence under similar conditions. Additionally both SL1 and commercial lipase (Lipolase^®^) present a similar stability towards Lav+ liquid detergents used after 1 h incubation at 50°C (Figure 4B). These results show clearly that SL1 can be used in both liquid and solid detergents formulations.

## Conclusion

This work describes the characterization of the crude enzymatic preparation containing a lipase produced by a novel *Staphylococcus *sp strain. The lipase preparation shows a high activity and stability in high alkaline pH and high temperatures. It showed stability not only towards the non-ionic surfactants like Triton X-100 and Tween 20, but also towards the strong anionic surfactant, SDS and oxidizing agents. Furthermore, the crude enzyme exhibited a high stability in the presence of various commercial liquid and solid laundry detergents. Considering its promising properties, one can say that SL1 can be considered as a potential candidate to be used as in biotechnology and essentially for application in the detergent industry.

## Competing interests

The authors declare that they have no competing interests.

## Authors' contributions

SC, SM and FH designed the experiments, analyzed the data and drafted the manuscript. SA and SS conceived research and approaches and have given final approval of the manuscript to be published. All authors read and approve the final manuscript.

## References

[B1] Grant WD Heaphy S Metagenomics and recovery of enzyme genes from alkaline saline environments Environ Technol 2010 31 1135 1143 10.1080/09593331003646661 20718296

[B2] Jiang Y Zhou X Chen Z Cloning, expression, and biochemical characterization of a thermostable lipase from *Geobacillus stearothermophilus* Environ Technol 2010 31 1107 1114 10.1080/09593331003677872 20718293

[B3] Guncheva M Zhiryakova D Radchenkova N Kambourova M Properties of Immobilized lipase from *Bacillus stearothermophilus *MC7. Acidolysis of triolein with caprylic acid World J Microbiol Biotechnol 2010 25 727 731

[B4] Abdelkafi S Ogata H Barouh N Fouquet B Lebrun R Pina M Scheirlinckx F Villeneuve P Carrière F Identification and biochemical characterization of a GDSL-motif carboxylester hydrolase from *Carica papaya *latex Biochim Biophys Acta 2009 1791 1037 1046 19555778 10.1016/j.bbalip.2009.06.002

[B5] Dharmaraj S Marine *Streptomyces *as a novel source of bioactive substances World J Microbiol Biotechnol 2010 26 2123 2139 10.1007/s11274-010-0415-6

[B6] Lee HK Lee JK Kim MJ Lee CJ Immobilization of lipase on single walled carbon nanotubes in ionic liquid Bull Korean Chem Soc 2010 31 650 652 10.5012/bkcs.2010.31.03.650

[B7] Mitidieri S Martinelli AHS Schrank A Vainstein MH Enzymatic detergent formulation containing amylase from *Aspergillus niger*: a comparative study with commercial detergent formulations Bioresour Technol 2006 97 1217 1224 10.1016/j.biortech.2005.05.022 16112858

[B8] Jeon JH Kim JT Kim YJ Kim HK Lee HS Kang SG Kim SJ Lee JH Cloning and characterization of a new cold-active lipase from a deep-sea sediment metagenome Appl Microbiol Biotechnol 2009 81 865 874 10.1007/s00253-008-1656-2 18773201

[B9] Wang YX Srivastava KC Shen GJ Wang HY Thermostable alkaline lipase from a newly isolated thermophilic *Bacillus*, strain A30-1 (ATCC 53841) J Ferment Bioeng 1995 79 433 438 10.1016/0922-338X(95)91257-6

[B10] Fendri I Chaari A Dhouib A Jlassi B Carriere F Sayadi S Abdelkafi S Isolation, Identification and characterization of a new lipolytic *Pseudomonas *sp., from Tunisian soil Environ Technol 2010 31 87 95 10.1080/09593330903369994 20232682

[B11] Pinzon-Martinez DL Rodriguez-Gomez C Minana-Galbis D Carrillo-Chavez JA Valerio-Alfaro G Oliart-Ros R Thermophilic bacteria from Mexican thermal environments: isolation and potential applications Environ Technol 2010 31 957 966 10.1080/09593331003758797 20662384

[B12] Cherif S Gargouri Y Thermoactivity and effects of organic solvents on digestive lipase from hepatopancreas of the green crab Food Chem 2009 116 82 86 10.1016/j.foodchem.2009.02.009

[B13] Abdelkafi S Fouquet B Barouh N Durner S Pina M Scheirlinckx F Villeneuve P Carrière F *In vitro *comparisons between *Carica papaya *and pancreatic lipases during test meal lipolysis: potential use of CPL in enzyme replacement therapy Food Chem 2009 115 488 494 10.1016/j.foodchem.2008.12.043

[B14] Cherif S Frikha F Gargouri Y Miled N Fatty acid composition of green crab (*Carcinus mediterraneus*) from the Tunisian Mediterranean coasts Food Chem 2008 111 930 933 10.1016/j.foodchem.2008.05.007

[B15] Bradford MM A rapid and sensitive method for the quantitation of microgram quantities of protein utilizing the principle of protein-dye binding Anal Biochem 1976 72 248 254 10.1016/0003-2697(76)90527-3 942051

[B16] Gupta R Saroop J Jain S Effect of cultural and assay conditions on cell bound lipase from a bacterial isolate SJ-15 Asian J Microbiol Biotechnol Environ Sci 2004 6 151 154

[B17] Ferrato F Carriere F Sarda L Verger R A critical revaluation of the phenomenon of interfacial activation Methods Enzymol 1997 286 327 346 9309657 10.1016/s0076-6879(97)86018-1

[B18] Simons JW Adams H Cox RC Dekker N Gotz F Slotboom AJ Verheij HM The lipase from *Staphylococcus aureus*. Expression in *Escherichia coli*, large-scale purification and comparison of substrate specificity to *Staphylococcus hyicus *lipase Eur J Biochem 1996 242 760 769 10.1111/j.1432-1033.1996.0760r.x 9022707

[B19] Sayari A Agrebi N Jaoua S Gargouri Y Biochemical and molecular characterization of *Staphylococcus simulans *lipase Biochimie 2001 83 863 871 10.1016/S0300-9084(01)01327-X 11698108

[B20] Mosbah H Sayari A Mejdoub H Dhouib H Gargouri Y Biochemical and molecular characterization of *Staphylococcus xylosus *lipase Biochim Biophys Acta 2005 1723 282 291 10.1016/j.bbagen.2005.03.006 15837431

[B21] Rosenstein R Gotz F Staphylococcal lipases: Biochemical and molecular characterization Biochimie 2000 82 1005 1014 10.1016/S0300-9084(00)01180-9 11099797

[B22] Horchani H Mosbah H Ben Salem N Gargouri Y Sayari A Biochemical and molecular characterisation of a thermoactive, alkaline and detergent-stable lipase from a newly isolated *Staphylococcus aureus *strain J Mol Cat B 2009 56 237 245 10.1016/j.molcatb.2008.05.011

[B23] Chen SJ Cheng CY Chen TL Production of an alkaline lipase by *Acinetobacter radioresistens* J Ferment Bioeng 1998 86 308 312 10.1016/S0922-338X(98)80135-9

[B24] Illanes A Stability of biocatalysts J Biotechnol 1999 2 7 15

[B25] Abdelkafi S Sayadi S Ben Ali Gam Z Casalot L Labat M Bioconversion of ferulic acid to vanillic acid by *Halomonas elongata *isolated from table-olive fermentation FEMS Microbiol Lett 2006 262 115 120 10.1111/j.1574-6968.2006.00381.x 16907747

[B26] Schmidt M Larsen DM Stougaard P A lipase with broad temperature range from an alkaliphilic *gamma-proteobacterium *isolated in Greenland Environ Technol 2010 31 1091 1100 10.1080/09593331003770289 20718291

[B27] Haki GD Rakshit SK Developments in industrially important thermostable enzymes: a review Bioresour Technol 2003 89 17 34 10.1016/S0960-8524(03)00033-6 12676497

[B28] Elkhattabi M van Geldr P Bitter W Tommassen J Role of the calcium ion and the disulfide bond in the *Burkholderia glumae *lipase J Mol Cat B 2003 22 329 338 10.1016/S1381-1177(03)00047-X

[B29] Noble MEM Cleasby A Johnson LN Egmond MR Frenken LGJ The crystal structure of triacylglycerol lipase from *Pseudomonas glumae *reveals a partially redundant catalytic aspartate FEBS Letters 1993 331 123 128 10.1016/0014-5793(93)80310-Q 8405390

[B30] Tiesinga JW van Pouderoyen G Nardini M Ransac S Dijkstra BW Structural basis of phospholipase activity of *Staphylococcus hyicus *lipase J Mol Biol 2007 371 447 456 10.1016/j.jmb.2007.05.041 17582438

[B31] Gupta R Beg O Lorenz P Bacterial alkaline proteases: molecular approaches and industrial applications App Microbiol Biotechnol 2002 59 15 32 10.1007/s00253-002-0975-y 12073127

[B32] Outtrup H Dambmann C Christiansen M Aaslyng DA *Bacillus *sp. JP395, method of making and detergent composition, US Patent number 1995 55 466 594

[B33] Wolff AM Showell MS Venegas MG Barnett BL Wertz WC Laundry performance of subtilisin protease, in: R.C. Bott (Ed.), Subtilisin Enzymes: Practical Protein Engineering Plenum Press, New York 1996 113 120 10.1007/978-1-4613-0319-0_12 8796315

[B34] Tsuchiya K Nakamura Y Sakashita H Kimura G Purification and characterization of a thermostable alkaline protease from alkalophilic Thermoactinomyces sp. HS682 Biosci Biotechnol Biochem 1992 56 246 250 10.1271/bbb.56.246 1368301

